# Oral Ferrous Sulphate Improves Functional Capacity on Heart Failure Patients with Iron Deficiency Anemia

**DOI:** 10.5334/gh.1151

**Published:** 2022-11-22

**Authors:** Lita Dwi Suryani, Sunu Budhi Raharjo, Rima Sagita, Hansen Angkasa, Yosafat Lambang Prasetyadi, Franciscus D. Suyatna, Nani Hersunarti, Rarsari Soerarso, Bambang Budi Siswanto, Hary Sakti Muliawan

**Affiliations:** 1 Department of Cardiology and Vascular Medicine, Faculty of Medicine, Universitas Indonesia/National Cardiovascular Center Harapan Kita Hospital, Indonesia/Universitas Indonesia Hospital, ID; 2 Department of Pharmacology and Therapeutic, Faculty of Medicine, Universitas Indonesia, ID

**Keywords:** Heart failure, Iron deficiency anemia, Oral ferrous sulphate, Functional capacity

## Abstract

**Background:**

Iron deficiency anemia (IDA) in heart failure (HF) is associated with poor functional capacity. Several studies reported the benefit of iron therapy in HF with IDA on improving functional capacity. Therefore, we attempt to investigate the effect of oral iron supplementation on functional capacity in HF patients with IDA.

**Results:**

A double blind randomized controlled trial was conducted in National Cardiovascular Center Harapan Kita Hospital Universitas Indonesia. A total of 54 HFREF patients with IDA were enrolled and randomized to either oral Ferrous Sulphate (FS) 200 mg three times a day or placebo with 1:1 ratio for 12 weeks. Primary outcome was functional capacity measured by a six-minute walk test. There were 41 participants completed the study (FS n = 22, placebo n = 19). Ferrous sulphate significantly improved functional capacity changes (46.23 ± 35 m vs –13.7 ± 46 m, p < 0.001, CI –86.8 to –33.2) compared with placebo groups respectively after 12 weeks intervention.

**Conclusions:**

Oral FS supplementation for 12 weeks significantly improved functional capacity in HF patients with IDA.

**Trial registration:**

clinicaltrials.gov, NCT02998697. Registered 14 December 2016 – Retrospectively registered, https://clinicaltrials.gov/ct2/show/NCT02998697.

## Introduction

Iron deficiency anemia (IDA) is one of the most common nutritional deficiencies in the world affecting approximately 15% global population and becoming a common co-morbidity in heart failure (HF) patients [1]. It is estimated that nearly 50% of HF patients have Iron deficiency regardless of anemia [2,3]. Although the 2016 ESC heart failure guideline defined absolute ID as serum ferritin <100 ng/ml and functional ID as serum ferritin 100–299 ng/ml with transferrin saturation <20% [4]. The ID with or without anemia as HF comorbidity the independent predictor of mortality. Moreover, anemia in HF patient is also associated with lower functional capacity and quality of life [3,4,5,6,7].

Many studies have investigated that treating the anemia condition could give a positive effect for the outcome. One of the proven drugs of choice for treating this condition is intravenous (IV) iron supplement. This drug improved haemoglobin, ferritin, transferrin saturation and functional capacity [8,9]. However, intravenous iron supplement is not readily available in low-to-middle income countries such as Indonesia. The main reason was the expensive IV iron regimen was not covered by National Health Insurance. Hence, there is a need to obtain a cheaper, more available alternative.

Oral iron supplementation such as ferrous sulphate tablets is readily available and is cheaper compared to IV iron supplement. (Table 1) However, recent opinion suggests that inflammation in HF patients can induce hepcidin level which inhibits iron absorption bioavailability [10,11,12,13]. Confronting this opinion, Niehaus et al. showed that 130 mg of oral ferrous sulphate supplementation improved iron levels in HF patients [14]. Therefore, this study investigates the effectiveness of oral iron, especially ferrous sulphate on improving functional capacity in HFREF patients with IDA on Indonesia population. The importance of this study was finding the potency of a widely available oral iron regimen in Indonesia in the form of ferrous sulphate for heart failure with reduced ejection fraction.

**Table 1:** Comparison of Oral vs Intravenous Iron.


	ORAL IRON	INTRAVENOUS IRON

PRICE	INEXPENSIVE	EXPENSIVE

Administration	Administered everywhere	In-hospital administration

Efficacy in Heart Failure	Inconclusive efficacy	High efficacy

Adherence	Low adherence due to high intake frequency	High adherence due to single administration

Availability in Developing Country	Highly available	Rarely available


## Methods

This study was designed as a single centre, randomized, double blind, placebo-controlled trial. It was approved by the institutional review board in National Cardiovascular Center Harapan Kita Hospital Universitas Indonesia (NCCHK-UI). This study was registered at Clinicaltrials.gov with registration number NCT 02998697. All of the co-authors were involved throughout the study conduct. All participants who eligible for the study gave their written consent.

This study was conducted in NCCHK-UI outpatient clinic from January until November 2016. The participants were selected based on following inclusion and exclusion criteria. Inclusion criteria were age 18–75 years old, HFREF <45% with NYHA functional class II–III who receives guideline directed medical therapy, haemoglobin (Hb) <13 g/dl for men and <12 g/dl for women, ferritin <100 ng/ml or between 100–300ng/ml with transferrin saturation <20%, eGFR >30 ml/min/1.73 m^2^, and willing to give written consent to join the study. Exclusion criteria were active bleeding, infection, malignancy, haematology disorder, peptic ulcer, intolerant to ferrous sulphate, received intravenous iron within past month, Implanted permanent pacemaker, intracardiac defibrillator, or cardiac resynchronization therapy, NT-pro BNP level >4000 pg/ml, liver disease with SGOT/SGPT >3x normal value, congenital heart disease, valvular heart disease, right heart failure due to pulmonary hypertension, underwent primary percutaneous intervention or coronary arterial bypass operation in the last three months, stroke or transient ischemic attack in the last three months.

Participants with HFREF and IDA who fulfilled inclusion and exclusion criteria were enrolled and randomized to receive either placebo or ferrous sulphate 200 mg three times a day (Kimia Farma, Indonesia) for 12 weeks. The subject allocation for each group was also randomized and concealed. Both of the participant and the investigator were blinded of the treatment. Before treatment initiation, the participant underwent baseline examination including clinical data, echocardiography, six-minute walk test (6MWT), and laboratory test such as iron profile, liver function, kidney function, and NT-pro BNP. Clinical follow up was performed every month in which we recorded clinical condition, pill counting, and side effect. Adverse events were observed and recorded within 12 weeks intervention period. After 12 weeks therapy, the participant underwent the same set of examination like the baseline for outcomes analysis.

The primary outcome was the differences of functional capacity measured by 6MWT after 12 weeks therapy of ferrous sulphate or placebo groups. Secondary outcomes including the differences of clinical outcomes, echocardiography parameters of left ventricular ejection fraction (LVEF), and laboratory results such as iron profile changes after 12 weeks intervention.

The type I error was set two-sided 0.05 with power of 80%. A minimal of 40 patients with HFREF and IDA would be randomized into 1:1 ratio and concealed to ferrous sulphate or placebo allocation for 12 weeks. A two-sided p value <0.05 was considered statistically significant. Baseline demographic, clinical, echocardiography, 6MWT, and laboratory examination including iron profile, liver function, kidney function, and NT-pro BNP were compared between placebo and ferrous sulphate group at baseline. Continuous and categorical data with normal distribution were displayed as mean ± standard error mean and percentage respectively. Abnormal continuous data distribution was displayed as median. Categorical data was displayed as percentage. Categorical data was analyzed using Chi square test. Numerical data was analyzed using independent t-tests with Welch’s correction for normal distribution or Mann Whitney test for abnormal distribution data. Analyses were performed using GraphPad prism version 8.3 (GraphPad Software. San Diego, CA 9218).

## Results

**Figure 1:**
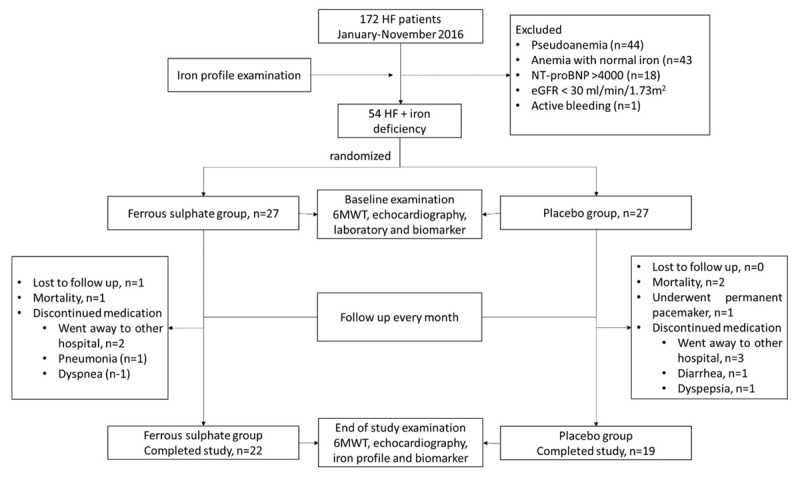
Consort diagram flow of this study. HF: Heart failure, eGFR: estimated glomerular filtration rate, 6MWT: 6-minute walk test.

We enrolled 172 HFREF patients from January until November 2016 (Figure 1). A total of 54 participants who fulfilled HFREF and IDA inclusion and exclusion criteria were randomized to receive ferrous sulphate or placebo group with 1:1 ratio. We observed three mortality events with 1 mortality on ferrous sulphate group and 2 mortality events on placebo group. One participant was loss to follow-up on ferrous sulphate group. Four participants from ferrous group discontinued their medication with 2 participants went to other hospital, 1 suffered from pneumonia infection, and 1 participant complained worsened dyspnoea. Five participants from placebo group discontinued their medication with 1 participant excluded due to pacemaker implantation, 3 participants went to other hospital, 1 participant complained worsened dyspnoea, and 1 participant complained worsened diarrhea. In the end, a total of 41 participants completed the study with 22 participants on ferrous sulphate and 19 participants on placebo.

Baseline characteristics of those groups were presented in Table 2. Overall, we did not find significant baseline difference between both groups. The mean age was 58 ± 9 and 57 ± 10 years old for ferrous sulphate and placebo group respectively. Both groups have NYHA functional class II–III. The mean LVEF was 34 ± 9% and 35 + 11% for ferrous group and placebo respectively. Functional capacity for both groups were 300 ± 85 and 309 ± 75 meter for ferrous sulphate and placebo group respectively. Level of mean haemoglobin on ferrous group was 11.6 ± 1.8 g/dl while 11.3 ± 1 for g/dl for placebo. Serum ferritin level was preserved >100 ng/dl for both groups with 121 ± 108 ng/ml on ferrous sulphate group and 110 ± 72 ng/ml on placebo. Both groups received similar heart failure guideline directed medical therapy.

**Table 2:** Baseline characteristics.


VARIABLE	GROUP/CATEGORY		P VALUE

	FERROUS SULPHATE (N = 27)	PLACEBO (N = 27)	

Gender			

Male	15 (68.2%)	11 (57.9%)	0.3

Female	7 (31.8%)	8 (42.1%)	0.3

Age (y.o)	58 ± 9	57 ± 10	0.7

BMI (kg/m^2^)	23.90 ± 3.62	22.87 ± 2.88	0.8

Hypertension	19 (86.4%)	16 (84.2%)	0.6

Diabetes	13 (59.1%)	11 (57.9%)	0.9

ADHF hospitalization	17 (77.3%)	16 (84.2%)	0.4

ACS hospitalization	11 (50%)	13 (68.4%)	0.3

Stroke	2 (9.1%)	3 (15.8%)	0.5

Gastrointestinal bleeding	2 (9.1%)	2 (10.5%)	0.9

Gastritis	4 (18.2%)	4 (21.1%)	0.8

History of CABG	4 (18.2%)	3 (15.8%)	0.8

History of PCI	10 (45.5%)	11 (57.9%)	0.4

Coronary Disease			

Normal Coronary	2 (9.1%)	2 (10.5%)	0.18

1 Vessel disease	3 (13.6%)	7 (36.8%)	

2 Vessel disease	3 (13.6%)	1 (5.3%)	

3 Vessel disease	11 (50%)	6 (31.6%)	

Not revascularize	3 (13.6%)	3 (15.8%)	

Atrial Fibrillation	4 (16.7%)	0 (0%)	0.09

Systolic BP (mmHg)	127 ± 22	118 ± 19	0.06

Diastolic BP (mmHg)	70 ± 13	70 ± 15	0.8

Peripheral pulse (x/min)	79 ± 15	82 ± 16	0.9

LVEF (%)	34 ± 9	35 ± 11	0.7

TAPSE (cm)	1.82 ± 0.48	1.80 ± 0.45	0.9

6MWT (m)	300 ± 85	309 ± 75	0.7

NYHA Functional Class			0.3

I	0 (0%)	0 (0%)	

II	11 (50.0%)	11 (57.9%)	

III	11 (50%)	8 (42.1%)	

IV	0 (0%)	0 (0%)	

Haemoglobin (g/dL)	11.6 ± 1.8	11.3 ± 1.0	0.6

Ferritin (ng/mL)	121 ± 108	110 ± 72	0.6

Transferin saturation (%)	15.6 ± 5	17 ± 7.6	0.4

eGFR (mL/min)	57 ± 30	51 ± 26	0.4

NT-pro BNP (pg/mL)	2810 ± 3116	3105 + 2354	0.7

Lactic acid (mmol/L)	1.4 ± 0.6	1.3 ± 0.6	0.4

SGOT (U/L)	18 ± 9	19 ± 6.6	0.6

SGPT (U/L)	18.6 ± 10.7	20 ± 13	0.6

eGFR	50.9 ± 26.2	57.4 ± 29.5	0.4

Haematocrit (%)	37.30 ± 5.41	33.58 ± 2.97	0.3

Relative reticulocyte (%)	1.3 ± 0.50	1 ± 0.43	0.2

Erythrocyte (10^6^/µL)	4.4 ± 0.83	4.10 ± 0.57	0.6

Leukocyte (10^3^/µL)	7.927 ± 2.2	7.938 ± 2.1	0.9

Platelet (10^3^/µL)	294 ± 609	270 ± 736	0.2

RDW (%)	14.3 ± 1.8	15 ± 1.8	0.2

MCV (fL)	84 ± 6.2	83 ± 8.0	0.8

MCH (pg)	28 ± 2.2	28 ± 3.5	0.9

MCHC (g/dL)	33 ± 1.0	33 ± 1.4	0.5

Medication			

– Diuretic	21 (95.5%)	16 (84.2%)	0.2

– ACE-I/ARB	22 (100%)	19 (100%)	1

– Beta Blocker	18 (81.8%)	18 (94.7%)	0.2

– MRA	9 (40.9%)	6 (31.6%)	0.5

– Statin	20 (90.9%)	18 (94.7%)	0.6

– Antiplatelet	20 (90.9%)	15 (78.9%)	0.3

– Anticoagulant	2 (9.1%)	4 (21.1%)	0.3

– Digitalis	2 (9.1%)	2 (10.5%)	0.9


No significant baseline differences between groups. Abbreviation: BMI = body mass index, ADHF = acute decompensated heart failure, ACS = acute coronary syndrome, eGFR = estimated glomerular filtration rate, RDW = red cell distribution width, MCV = mean corpuscular volume, MCH = mean corpuscular haemoglobin, MCHC = mean corpuscular haemoglobin concentration, ACE-I = Angiotensin II converting enzyme inhibitor, ARB=Angiotensin II receptor blocker.

**Figure 2:**
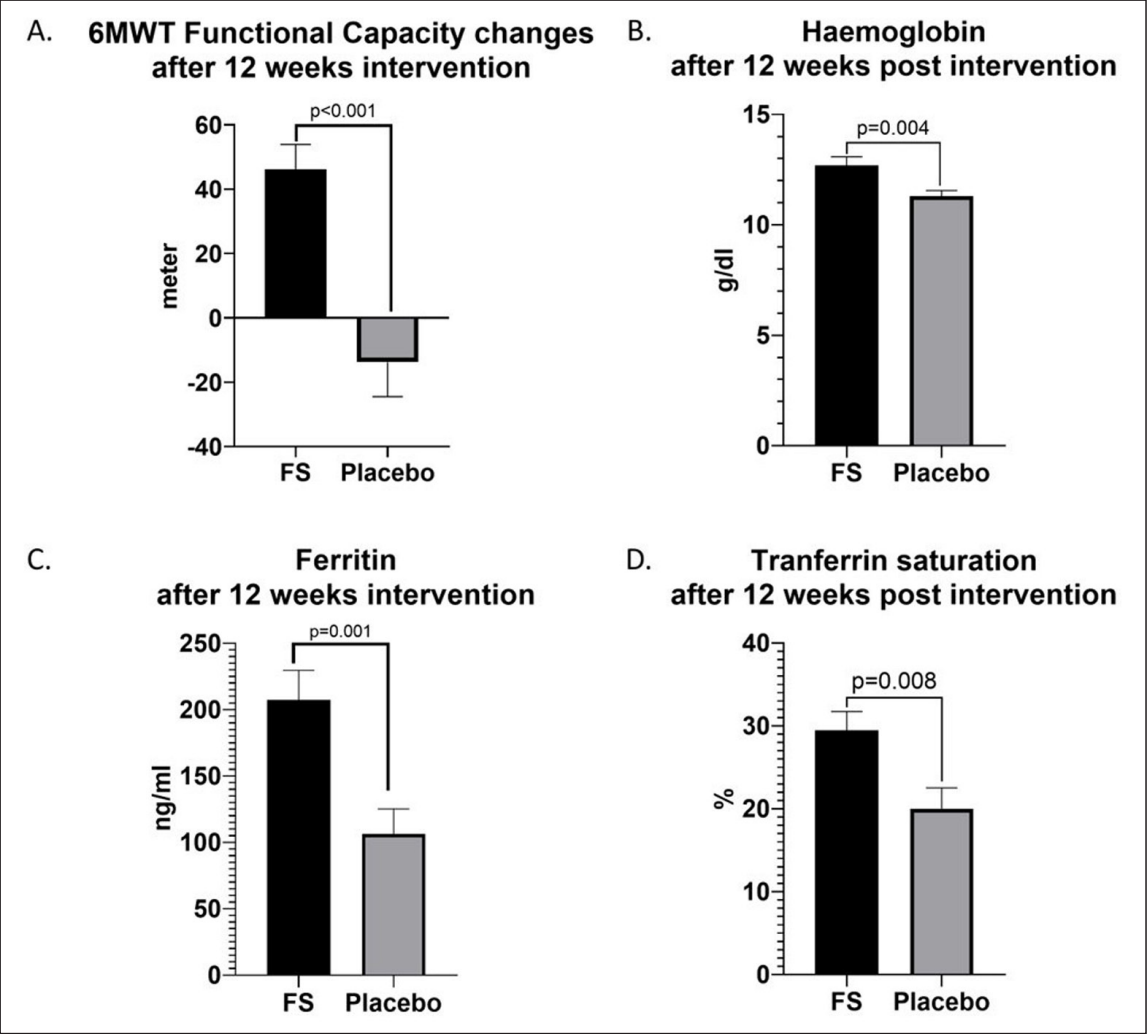
6MWT functional capacity, haemoglobin, and iron profiles after 12 weeks intervention. **A.** Significant functional capacity improvement in FS group compare to placebo. **B–D.** Ferrous sulphate significantly increase haemoglobin, ferritin, and transferrin saturation, and levels respectively compared to placebo.

We found a significant 6MWT functional capacity improvement in ferrous sulphate compared to placebo group after 12 weeks therapy (46.23 ± 35 m vs –13.7 ± 46 m, p < 0.001, CI –86.8 to –33.2, Figure 2A). Following 12 weeks intervention, we discovered a significant increase of haemoglobin levels in ferrous sulphate compared with placebo group (12.7 ± 1.8 g/dl vs 11.3 ± 1 g/dl, p = 0.004, CI –2.3 to –0.4, Figure 2B). We also observed a significantly higher ferritin levels in ferrous sulphate compared with placebo group (207.3 ± 104 ng/ml vs 111.7 ± 81.4 ng/ml, p = 0.001, CI –160 to –41.87, Figure 2C). Lastly, there was also a substantial TSAT increased in Ferrous group compared to placebo (29.5 ± 10.4% vs 20 ± 10.6%, p = 0.008, CI –16.3 to –2.6, Figure 2D). We presented the baseline and after 12-weeks follow up of the primary output in Table 3.

**Table 3:** Primary Outcome Baseline and 12-Weeks Follow Up.


VARIABLE	GROUP/CATEGORY			

	FERROUS SULPHATE (N = 27)		PLACEBO (N = 27)	
	
	BASELINE	12-FU	BASELINE	12-FU

6MWT Functional Capacity	300 ± 85	349 ± 86	309 ± 75	305 ± 85

Haemoglobin	11.6 ± 1.8	12.6 ± 1.8	11.3 ± 1.0	11.2 ± 1

Ferritin	121 ± 108	207 ± 106	110 ± 72	112 ± 83

Transferrin Saturation	15.6 ± 5	28 ± 10	17 ± 7.6	20 ± 11

NT-pro BNP	2810 ± 3116	1645 ± 1289	3105 ± 2354	1262 (128 – 7424)^a^

LVEF	34 ± 9	37 ± 10	35 ± 11	34 ± 12


^a^ = presented in median (min – max) due to abnormal data. Abbreviation: 6MWT = 6-minute walk test, NT-pro BNP = N Terminal pro Brain Natriuretic Peptides, LVEF = left ventricular ejection fraction.

We observed a significant NYHA functional class improvement after 12 weeks intervention in ferrous sulphate group compared with placebo group after with p = 0.01 (Table 4). There was no significant difference of adverse event between both groups during 12 weeks monitoring. We reported 1 HF rehospitalization on ferrous sulphate and 2 HF rehospitalization on placebo group. We found 1 mortality event on ferrous sulphate group and 2 mortality events on placebo group. Two severe gastrointestinal side effect on each group were discovered. In addition, we also recorded 5 minor gastrointestinal side effect on ferrous sulphate group and 7 minor gastrointestinal side effect on placebo group during 12 weeks monitoring. These adverse and side effect findings are summarized in Table 5.

**Table 4:** NYHA functional class after 12 weeks intervention.


NYHA FUNCTIONAL CLASS	FERROUS SULPHATE	PLACEBO	CHI SQUARE P VALUE

I	2 (9%)	0	0.001

II	18 (82%)	2 (10%)	

III	2 (9%)	17 (90%)	

Total	22	19	


Lower proportion of NYHA class III in ferrous sulphate compared to placebo group. Abbreviation: NYHA = New York Heart Association.

**Table 5:** Adverse event and gastrointestinal side effects.


EVENTS	FERROUS SULPHATE (N)	PLACEBO (N)	P VALUE

HF rehospitalization	1/27	2/27	0.4

Death	1/27	2/27	0.4

Severe gastrointestinal side effects	2/27	2/27	0.4

Minor gastrointestinal side effects	5/27	7/27	0.1

Total Events	9/27	13/27	


Abbreviation: HF= heart failure.

**Figure 3:**
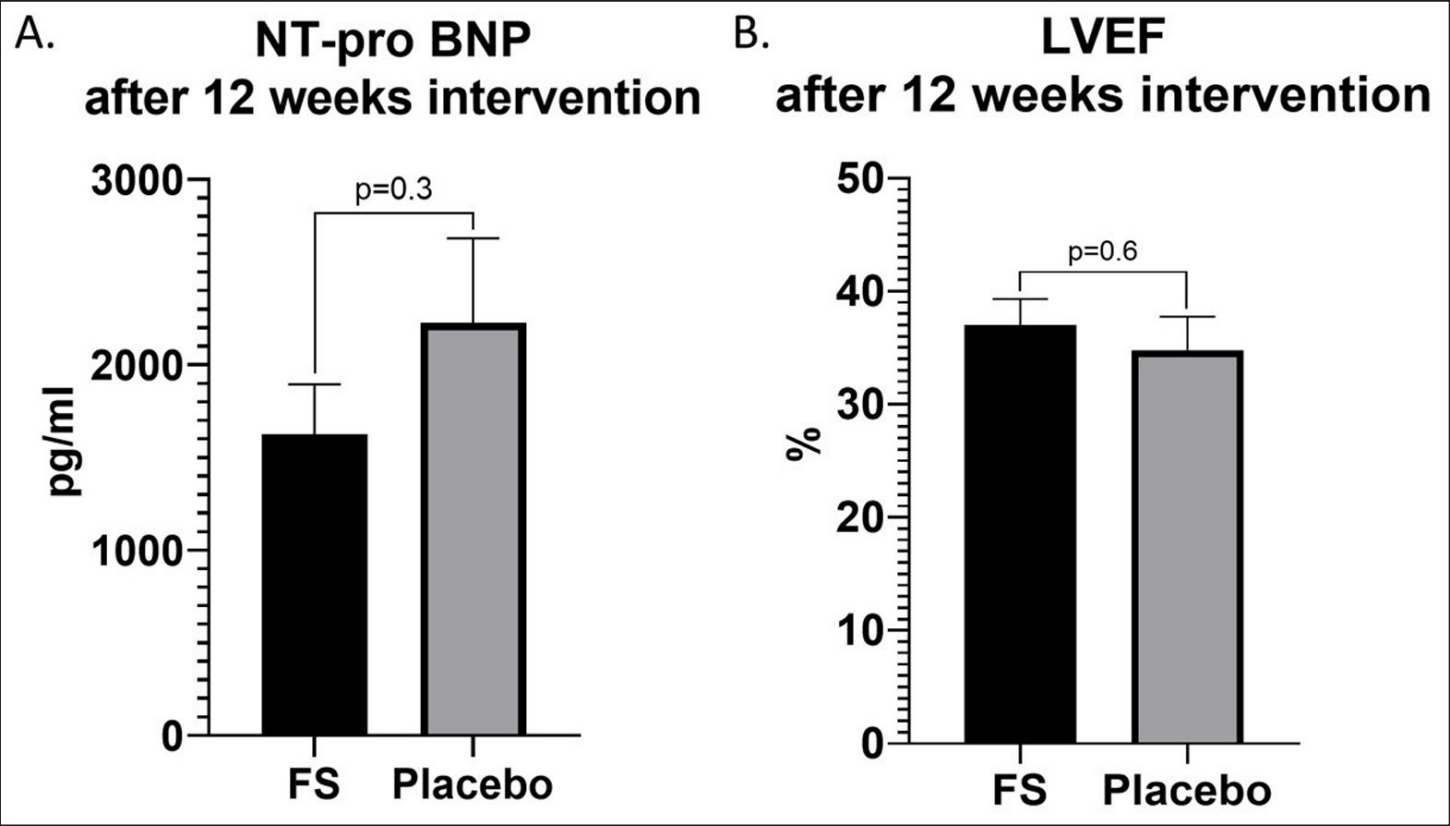
NT-pro BNP and LVEF after 12 weeks intervention. A–B. No significant NT-pro BNP and LVEF respectively between both groups after 12 weeks intervention.

We did not observe significant NT-pro BNP differences between both groups after 12 weeks intervention (ferrous sulphate 1625 ± 1233 pg/ml vs placebo 2055 ± 1931 pg/ml, p = 0.3, CI –478 to 1682, Figure 3A). We also did not find significant LVEF differences between both groups after 12 weeks intervention (ferrous sulphate 37 + 10.3% vs placebo 34.8 + 12.2%, p = 0.5, CI –9.8 to 5.4, Figure 3B).

## Discussion

We conducted a single centre, randomized double-blind, placebo-control trial to investigate the effect of oral iron supplementation in HFREF patients with IDA. We found significant improvement of functional capacity from 6MWT and NYHA functional class after 12 weeks ferrous sulphate treatment compared to declining functional capacity in placebo group. In addition, ferrous sulphate treatment for 12 weeks was able to increase haemoglobin, ferritin and TSAT levels. Overall, ferrous sulphate is well tolerated without any significant adverse event differences between both groups.

Our study demonstrated the benefit of ferrous sulphate 200 mg daily three times a day for 12 weeks on improving functional capacity and restoring iron profiles. We observed 0.9 g/dl Hb, 69% ferritin, 88% TSAT increment which comparable to IV iron supplementation studies such as FAIR-HF and CONFIRM-HF [8,15]. In accordance, our 6MWT functional capacity improvement was also comparable with those studies supporting the benefit of iron profile restoration in HF patients with anemia due to ID. The possible explanation on why the oral iron supplementation on our study could improve the functional capacity was because oral ferrous sulphate can be absorbed better the other oral iron regimen. Despite improvement of functional capacity in our study, we could not identify significant amelioration on NT-pro BNP and LVEF. We think that the time span in this study was too short to induce substantial recovery in myocardial function.

Iron profiles and haemoglobin restoration in our study suggest the occurrence of iron absorption through gastrointestinal tract. Previous studies assumed that oral iron supplementation was not efficient in HF patient with IDA due to high hepcidin level [16,17,18,19]. However, the role of hepcidin in HF with IDA is still debatable as there are conflicting results. Elevated hepcidin levels were observed in HF patients with mild symptom NYHA functional class I–II [19]. However, the elevation of hepcidin in this study was not correlated with IL-6 which was considered as inflammatory cytokines that increased hepcidin level and reduced GI iron absorption. Hence, high ferritin on this study suggests that hepcidin elevation was secondary due to high ferritin. Furthermore, low hepcidin level was exhibited in advanced HF [19,20,21]. Previous studies showed that dilutional anemia and elevated erythropoietin in more severe HF might decrease hepcidin levels and nullify the effect of inflammation on hepcidin [20,21]. Our HF population displayed functional IDA with moderate NYHA functional class II–III and elevated NT-pro BNP.

Contradictive oral iron supplementation in HFREF with IDA result was reported by IRONOUT HF study [22]. They observed no significant ferritin, TSAT, and 6MWT functional capacity changes after 16 weeks iron polysaccharide supplementation 150 mg twice daily. These conflicting results might be explained by the differences of oral iron types. Iron polysaccharide was known to be more tolerable compared to ferrous sulphate [23]. However, our study did not show significant adverse effect differences between ferrous sulphate and placebo group. Recent studies demonstrated higher iron restoration on ferrous group compared to iron polysaccharide group without significant side effect differences [23,24,25].

As mentioned earlier, the conflicting results of this study and the previous ones might be explained by the differences in oral iron types. In this study, the oral ferrous sulfate was indicated to have better efficacy than ferrous polysaccharide. In the BESTIRON study, a randomized, double-blind single-center trial designed to compare the effectiveness of an iron polysaccharide complex vs. ferrous sulfate for the treatment of nutritional ID anemia in infants and young children, the ferrous sulphate resulted in a more significant increase in hemoglobin concentration at 12 weeks [26]. Furthermore, In IRON-HF study, both ferrous sulphate or IV iron sucrose supplementation for five weeks in HFREF patients with IDA was significantly able to increase ferritin and TSAT levels [9]. Although, no functional capacity improvement was observed on five weeks ferrous sulphate group in IRON-HF study. Our results suggest that at least 12 weeks ferrous sulphate treatment was needed to improve functional capacity in HF patients with IDA. Moreover, ferrous sulphate is cheaper and widely available in developing country such as Indonesia. Therefore, we think that ferrous sulphate can be used as an alternative to iron IV for restoring iron profile and improving functional capacity in HFREF patients with IDA.

## Limitation

Due to limited resources, we did not evaluate hepcidin which would provide excellent mechanistic insight on iron metabolism in our populations. Previous studies showed that higher hepcidin levels which often found in HF patients inhibited oral iron absorption thus limiting its bioavailability. Nevertheless, we found significant restoration of iron profiles which suggest oral iron GI absorption occurrence.

## Conclusions

Oral ferrous sulphate for 12 weeks significantly improved functional capacity and NYHA functional class in HFREF patients with IDA. Furthermore, oral ferrous sulphate for 12 weeks also restored haemoglobin and iron profiles. Therefore, oral ferrous sulphate remains a feasible alternative therapeutic option for HFREF patients with IDA in developing countries especially in Indonesia.

## Take Home Message

Oral ferrous sulphate has a huge potential as adjunctive therapy for heart failure patients with iron deficiency anemia, especially in developing countries like Indonesia. Further investigation in multiple centers with a bigger sample size will be very beneficial to gain a better conclusion regarding the topic.

## Data Accessibility Statement

The data will be available for the readers if requested for further iron supplementation in heart failure research.

## Additional File

The additional file for this article can be found as follows:

10.5334/gh.1151.s1 Supplementary Table. Iron-containing Indonesian Daily Food.

## Abbreviations

6MWT: 6-minute Walk Test

CI: Confidence Interval

eGFR: Estimated Glomerulus Filtration Rate

FS: Ferrous Sulphate

GI: Gastrointestinal

Hb: Hemoglobin

HF: Heart Failure

HFREF: Heart Failure with Reduced Ejection Fraction

IDA: Iron Deficiency Anemia

IV: Intravenous

LVEF: Left Ventricular Ejection Fraction

NCCHK-UI: National Cardiovascular Center Harapan Kita Hospital Universitas Indonesia

NT-pro BNP: N-Terminal Pro Brain Natriuretic Peptide

NYHA: New York Heart Association

SGOT: Serum Glutamic Oxaloacetic Transaminase

SGPT: Serum Glutamic Pyruvic Transaminase

TSAT: Transferrin Saturation
