# Supplementary material for: Oral Ferrous Sulphate Improves Functional Capacity on Heart Failure Patients with Iron Deficiency Anemia
Source: Glob Heart. 2022 Nov 22;17(1):81. doi: 10.5334/gh.1151 (PMC9695150; doi:10.5334/gh.1151)
Supplement: Supplementary Table. — Iron-containing Indonesian Daily Food. [file gh-17-1-1151-s1.pdf]

Supplementary Table. Iron-containing Indonesian Daily Food.[1]

Food Source	Iron/100 grams (mg)
Steamed spinach	15.8
Oyster	3.7
Clams	15.6
Beef	2.6
Tempe[a]	4.9
Chicken's liver	15.8
Beef liver	4.0

a= Indonesian local food of soybean fermentation

1. Asosiasi Keluarga Gizi. AKG FKM UI | 7 Sumber Makanan Kaya Zat Besi [Internet]. Akg.fkm.ui.ac.id. 2020 [cited 24 July 2022]. Available from: https://akg.fkm.ui.ac.id/7-sumber-makanan-kaya-zat-besi/
